# Altered Dynamic Postural Control during Step Turning in Persons with Early-Stage Parkinson's Disease

**DOI:** 10.1155/2012/386962

**Published:** 2012-01-29

**Authors:** Jooeun Song, Susan Sigward, Beth Fisher, George J. Salem

**Affiliations:** ^1^Jacquelin Perry Musculoskeletal Biomechanics Research Laboratory, Division of Biokinesology and Physical Therapy, University of Southern California, Los Angeles, CA 90089-9006, USA; ^2^Department of Neurology, Keck School of Medicine, University of Southern California, Los Angeles, CA 90089-9006, USA

## Abstract

Persons with *early-stage* Parkinson's disease (EPD) do not typically experience marked functional deficits but may have difficulty with turning tasks. Studies evaluating turning have focused on individuals in advanced stages of the disease. The purpose of this study was to compare postural control strategies adopted during turning in persons with EPD to those used by healthy control (HC) subjects. Fifteen persons with EPD, diagnosed within 3 years, and 10 HC participated. Participants walked 4 meters and then turned 90°. Dynamic postural control was quantified as the distance between the center of pressure (COP) and the extrapolated center of mass (eCOM). Individuals with EPD demonstrated significantly shorter COP-eCOM distances compared to HC. These findings suggest that dynamic postural control during turning is altered even in the early stages of PD.

## 1. Introduction

Postural control is the ability to alter the magnitude and patterns of segmental kinematics (e.g., trunk and limb movements) in order to direct body position in response to external mechanical demands imposed during static and dynamic tasks such as turning [1, 2]. Functional independence, and consequently quality of life, is compromised in individuals with postural control deficits. Persons with *early-stage* Parkinson's disease (EPD), Hoehn and Yahr stage 1 and 2, may not demonstrate overt clinical symptoms and may describe only minimal levels of functional impairment, such as reduced gait velocity and stride length, during simple movement tasks including straight walking [3, 4]. However, they often demonstrate altered postural control during standing tasks [5] and report difficulty with turning [6]. Turning difficulty becomes a sensitive indicator of a higher prevalence of freezing and falling in persons with *advance*d PD (Hoehn and Yahr stage ≥3 with moderate to severe symptoms) [7, 8].

Turning is a challenging task that aims to transport the body's mass in a new direction. It requires deceleration of the body's center of mass (COM; the position that represents the equilibrium point of the body's mass), rotation of the axial segments, and acceleration of the COM in the new direction [9, 10]. This is accomplished in three consecutive steps: approach, pivot, and acceleration steps embedded within two phases. During these phases individuals transition from double limb to single limb stance before returning to double limb stance [11]. Studies have established that in young, healthy individuals the redirection of the COM into the new direction of travel is initiated through appropriate foot placement during phase 1 (from approach step to pivot step). During phase 2 (from pivot step to acceleration step) trunk movements are used to control turning [12].

The demands of turning present unique challenges to individuals with impaired postural control as they are required to initiate a state of disequilibrium during single limb stance in order to change directions during an ongoing movement [9, 13]. This disequilibrium is created by increasing the distance between the body's COM and the center of pressure (COP; the equilibrium point of the distribution of the resultant ground reaction force applied to the base of support). An increased distance between these two points not only creates momentum necessary to turn, but also requires increased neuromuscular control (e.g., neural drive, muscle forces, and joint power) to redirect and control this momentum. Alterations in turning strategies are thought to reflect an individual's inability to meet these increased neuromuscular demands. For example, when compared to healthy controls, persons diagnosed with advanced PD utilize postural control strategies that include longer turning times [14] along with a greater number of smaller steps [8, 14] to complete a turn. These postural adjustments serve to decrease the body's momentum, reduce the distance between the COM and the COP, and in turn decrease the neuromuscular demands. While alterations in postural control strategies have been observed in individuals with advanced PD, they have not been characterized in individuals diagnosed with EPD.

Individuals diagnosed with EPD do report difficulty turning [6]. However, in contrast to individuals diagnosed with advanced PD, they do not frequently exhibit observable movement impairments that could impact turning such as shuffling gait, freezing episodes, and *en bloc* movements. [3, 4, 15, 16]. A more detailed evaluation of postural control strategies employed by individuals diagnosed with EPD during turning is needed. However, more traditional measures of postural control that relate the distance between the positions of the COP and COM may not be sensitive enough to detect differences between individuals diagnosed with EPD and healthy controls because they do not take into account the dynamic nature of the turning task. During dynamic tasks it is important to consider not only the position of the COM but also the magnitude and direction of the COM velocity in relation to the COP [17, 18].

Despite self-reports of difficulty turning in persons with EPD, studies to date have not characterized the postural control strategies used during turning in this cohort. Early identification of these strategies may be used to develop effective intervention protocols that (1) improve turning capabilities and (2) increase balance confidence in persons with EPD. Therefore, the purpose of this study was to characterize the differences in postural control during a step turning activity, between persons with EPD and healthy age-matched control (HC) participants. We hypothesized that, compared to HC participants, persons with EPD would demonstrate a dynamic postural control strategy that reduced the demands on the neuromuscular system. Specifically, we hypothesized that when accounting for the position, magnitude and velocity of the COM persons with EPD would demonstrate shorter distances between the COP and the eCOM than healthy controls during both phases of a step turn at 90 degrees. Moreover, this will be accomplished by both decreasing their COM velocity and the distance between their COP and the COM.

## 2. Methods

### 2.1. Participants

Fifteen persons with EPD and 10 HC subjects participated. Participant characteristics are provided in Table 1. A fellowship-trained movement disorder specialist confirmed diagnosis of idiopathic PD in our participants, performed the Unified Parkinson's Disease Rating Scale (UPDRS), and determined Hoehn and Yahr stage for each individual participant. Participants that had pharmacological treatment were stable and tested while they were on their routine therapy (Table 2). At the time of testing, none of the participants exhibited any fluctuations in motor ability throughout the day, dyskinesia, dystonia, or other signs of involuntary movement.

The inclusion criteria for the early PD group were the following: (1) age ≥18 years old, (2) able to ambulate at least 14 meters (time not measured) without a walker or other devices, (3) diagnosed with PD within 3 years [19], (4) Hoehn and Yahr stages 1-2 (indicating EPD), and (5) stable on PD medications. Healthy control participants were age and gender-matched to the participants in the early PD cohort. Participants were excluded from the study for the following: (1) surgical intervention for persons with PD, (2) Mini-Mental State Exam (MMSE) score <24 [20], (3) comorbidities affecting gait (e.g., diabetes, musculoskeletal injury, arthritis, vestibular disorders), (4) severe vision problems, and (5) pregnancy.

### 2.2. Protocol

**Figure 1:**
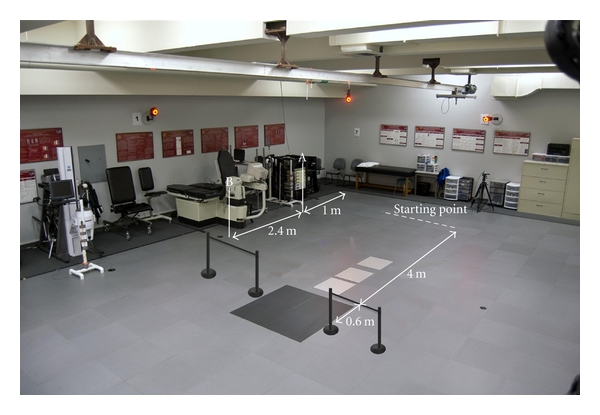
Laboratory setup. Dashed line: starting point; A: first trigger; B: second trigger; black square: force plate (AMTI 1.2 m × 1.2 m, 1560 Hz). Two stanchions were placed at the midpoint of each force plate. Starting point to A: 1 m. A to B: 2.4 m. B to force plate: 0.6 m. Force plate to stanchions: 0.6 m.

All testing took place in the Musculoskeletal Biomechanics Research Laboratory at the University of Southern California (USC). Procedures were explained to each participant and each participant signed an informed consent form approved by the Institutional Review Board of the USC. Participants were instructed to walk straight at a “self-selected, comfortable pace and turn at the designated stanchions at a right angle” toward their dominant leg and then continue walking in the new direction (Figure 1). Prior to testing the dominant leg was determined as the leg they would use to kick a ball as far as possible. No other instructions were given to the participants. The subjects started 1 meter from the first timing trigger. They then walked for an additional 2.4 meters before walking through the second timing trigger, which was located 0.6 meters in front of the force plate.

A total of 10 turning trials were recorded for each participant. The first three successful trials during which they used a *step turn* strategy were considered for analysis. A step turn is defined as a change in direction opposite to the pivot foot [10, 13]. Ninety-degree turns were selected for analysis because these types of turns are associated with the navigation of corridors, street corners, and other common walking activities. Moreover, Sedgman and colleagues reported that the majority of turns experienced during activities of daily living were between 76° and 120° [21].

Kinematic data were sampled at 60 Hz using a motion analysis system (Vicon 612, Oxford Metrics Ltd., Oxford, England). Reflective markers (14 mm spheres) were placed bilaterally on the skin over specific anatomical landmarks including the anterior, posterior, and lateral cranium, acromion processes, anterior and posterior shoulders, greater tubercles of humerus, medial and lateral humeral epicondyles, radial styloid process, ulnar head, third metacarpophalangeal joints, 7th cervical vertebrae, sternoclavicular notch, iliac crests, anterior superior iliac spines, posterior superior iliac spines, L5-S1 joint, medial and lateral femoral epicondyles, medial and lateral malleoli, first and fifth metatarsal heads, and first proximal/distal phalanx. Additionally, cluster markers were placed with a band over the upper arms, lower arms, thighs, shanks, and shoe heels. Reflective markers were identified manually within the VICON Workstation software and then imported into Visual 3D software (C-Motion, Rockville, MD). 3D marker coordinates were lowpass filtered at a cut-off frequency of 6 Hz.

Kinetic data were captured using 1.2 m × 1.2 m AMTI (Advanced Mechanical Technologies, Inc., Newton, MA, USA) force platform at 1560 Hz. The size of the platform allowed for quantification of ground reaction forces throughout the entire task. Kinematic and kinetic data were interfaced to the same microcomputer allowing for synchronization of data.

### 2.3. Data Analysis

Dynamic postural control during turning was quantified using the method previously described by Hof (1) [17]. It was defined as the difference between the COP and an extrapolated COM calculated to account for the position, magnitude and velocity of the COM:

(1) $$\text{Dynamic}\;\text{Postural}\;\text{Control}=\text{COP}-\left(\text{COG}+\frac{\text{COMvel}}{\surd \left(g/l\right)}\right).$$

The COP was determined from the forces and moments obtained from the force platform. The position of the total body COM was defined using the weighted sum of the COM of all 15-body segments. Based on Winter [22], instantaneous velocity of the total body COM (COMvel) was computed from the linear total body COM positions (COMpos):

(2) $$\text{COMvel}\;n=\frac{\left[\text{COMpos}\;n+1-\text{COMpos}\;n-1\right]}{\Delta t},$$

where, *n* is the event frame, and Δ*t* is the time between event frames.

The center of gravity (COG) represents the vertical projection of the body's COM. It was calculated based on the medial-lateral and the anterior-posterior locations of the COM. The COM velocity was divided by the natural frequency of the limb. The natural frequency was calculated as *√*(*g*/*l*) where *g* is the acceleration of gravity and *l* is the length of the leg measured from the ankle joint center to the COM. The extrapolated COM (eCOM) was calculated as sum of the COG and the new COM velocity term:

(3) $$\text{eCOM}=\left(\text{COG}+\frac{\text{COMvel}}{\surd \left(g/l\right)}\right).$$

**Figure 2:**
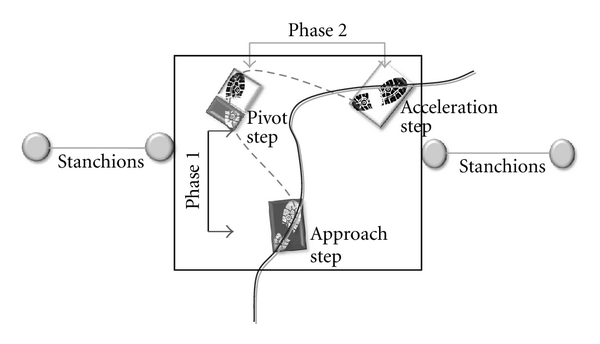
Schematic representation of the COP and the eCOM trajectories during the step turn. Solid line: eCOM trajectory; dashed gray line: COP trajectory; Phase 1: from approach step to pivot step; Phase 2: from pivot step to acceleration step.

The turning cycle was defined from heel strike of the approach step to heel strike of the acceleration step and was broken into 2 phases. Phase 1 was defined from heel strike of the approach step to heel strike of the pivot step. Phase 2 was defined from heel strike of the pivot step to heel strike of the acceleration step (Figure 2). The dependent variable dynamic postural control was measured as the peak distance between the COP and the eCOM. Peak distance between the COP and the COG, and the peak COM velocity were identified for each phase. These measures were considered in the case in which dynamic postural control differed between groups, as alterations in both position and velocity can affect this measure of dynamic postural control. The average approach gait velocity across three successful trials was calculated over the 2.4 meters between the first trigger (A) and the second trigger (B) during turning (Figure 1). The single- and double-limb gait cycle phases were determined using force plate contact and the vertical velocity of the virtual center of each foot [23].

### 2.4. Statistical Analysis

To determine if differences in our dependent variable, dynamic postural control, existed between persons with EPD and HC participants across turning phases, a 2 × 2 (group × phase) ANOVA was performed. In the case in which differences in dynamic postural control were found between groups, independent *t*-tests were performed to determine if group differences existed in the input variables used to calculate dynamic postural control, position of the COG relative to the COP, and the COM velocity within each phase. All statistical analyses were performed using SPSS 15.0 (Chicago, IL) with an alpha level set at 0.05.

## 3. Results

Participant characteristics and approach gait velocity are provided in Table 1. There were no significant group differences for age, height, weight, or approach gait velocity (*P* > 0.05). In the EPD group, average time since diagnosis was 18.2 ± 13.9 months, average H&Y score was 1.9 ± 0.3, and average UPDRS motor score was 21.2 ± 6.7. Average UPDRS gait and postural stability subscores were 0.1 ± 0.4 and 0.3 ± 0.5, respectively.

**Figure 3:**
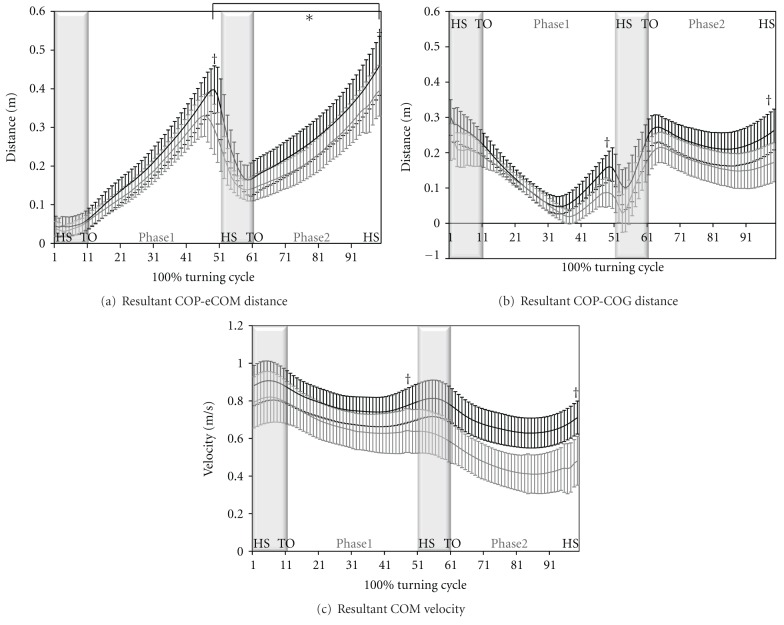
Resultant COP-eCOM distance (a), and resultant COP-COG distance (b), resultant COM velocity (c) between groups during the step turn cycle (±sd). HS: heel strike; TO: toe off; light gray line: persons with early-stage Parkinson's disease; black line: healthy control participants; gray shadow: double limb support time; white shadow: single limb support time. ^†^denotes statistically significant difference between *groups* (*P* < 0.05). *denotes statistically significant difference between *phases *(*P* < 0.05).

No significant group by phase interaction was found for dynamic postural control (*F* = 0.584, *P* = 0.453). Main effects of group and phase are found for dynamic postural control. Persons with EPD demonstrated statistically significant smaller peak COP-eCOM distances compared to HC participants during both Phase 1 (20.6% difference; 0.34 ± 0.05 versus 0.41 ± 0.06 m; *P* < 0.01) and Phase 2 (21.1% difference; 0.38 ± 0.06 versus 0.46 ± 0.07 m; *P* = 0.01) of the step turn (Figure 3(a)). The peak distance between the COP and the eCOM always occurred during single limb stance within each of the phases.

Compared to control participants, persons with EPD demonstrated statistically significant smaller peak COP-COG distances during both Phase 1 (30.8% difference; 0.13 ± 0.03 versus 0.17 ± 0.03 m; *P* < 0.01) and Phase 2 (28.6% difference; 0.21 ± 0.05 versus 0.27 ± 0.04 m; *P* < 0.05; Figure 3(b)).

Although there was no significant difference in the average approach gait velocity between groups, persons with EPD exhibited significantly slower peak COM velocity when compared with control participants during phase 1 (14% difference; 0.64 ± 0.10 versus 0.74 ± 0.10 m/s) and phase 2 (35% difference; 0.41 ± 0.11 versus 0.63 ± 0.08 m/s; *P* < 0.05; Figure 3(c)) of the turning cycle.

## 4. Discussion

This study identified differences in dynamic postural control strategies in persons with EPD during step turning activities compared to HC participants. Using the eCOM, we were able to account for not only the position of the COM but also the magnitude and velocity. This is particularly important during turning as the momentum of the COM is needed for forward propulsion and redirection. We found that persons with EPD utilized shorter distances between the COP and the eCOM during both phases of the turning cycle. For both phases the group differences were noted during single limb stance. This suggests that during a time in which postural control demands are greatest, individuals with EPD adopt a strategy that aims to decrease these demands. It is also important to note that the differences observed in postural control between the groups appear to be largely driven by alterations in magnitude, not timing, suggesting that individuals with EPD are not adopting an entirely new strategy but merely scaling the strategy typically used to turn (Figure 3).

Persons with EPD appeared to scale both position and velocity of the COM: factors used to calculate postural control in this study. Both the shorter peak distance between the COP and the COG, and the slower peak COM velocity exhibited by individuals with EPD during the turn limit the disequilibrium experienced by the individual. Both of these adjustments have the potential to decrease neuromuscular demands. A smaller COP-COG distance reduces the moment arm created for the body weight vector acting around the centers of joint rotation, and thus the magnitude of the muscular force required to control the COM [24]. Additionally, a slower COM velocity reduces the momentum of the COM and decreases the muscular force required to decelerate and redirect the COM.

These alterations are consistent with what has been observed in persons with more advanced PD (longer turning time and smaller steps) during turning [8, 14, 25]. The current data support self-reports of “difficulty in turning” from persons with EPD [6]. Our findings are similar to those reported by researchers investigating other transitional movement patterns in persons with more advanced PD, namely gait initiation and sit-to-walk activities. For example, Martin and colleagues [26] reported a shortening of the separation between the COM and the COP during gait initiation in persons with PD, compared to healthy older adults. Moreover, Buckley and colleagues [27] reported that compared to healthy control subjects, persons with PD utilized a conservative movement strategy that limited separation of COP-COM during sit-to-walk transitions. We demonstrated that when we challenged individuals with EPD with a turning task, alterations in postural control similar to those seen in more advanced stages were observed. This is of particular importance since this group does not commonly demonstrate obvious signs of gait disturbance [3, 4].

Although the current findings describe adjustments in postural control in individuals with EPD, they are not sufficient to tease out whether or not the COP-eCOM difference is a *primary deviation* or a *secondary compensation* of the disease. The reduced COM velocity demonstrated by our participants is in agreement with previous reports of slower turning velocity in persons with PD (i.e., task-specific bradykinesia) [14, 25]. As discussed, our data demonstrate that persons with EPD utilize a scaled motor control strategy that limits separation of the COP and the eCOM. This could be due to lack of neuromuscular control of the COM, limb and trunk position, or the result of bradykinesia or rigidity-*primary deviations* associated with compromised basal ganglia function. Alternatively, the findings may also be the result of *secondary compensation of the disease. *For example, this strategy could be adopted due to the inability to generate appropriate momentum or the presence of neuromuscular deficits, which limit adequate muscular force production [26, 28]. While we did not directly measure muscular force, our findings are consistent with reports of reduced lower extremity force generation in persons with PD [29]. In aggregate, the cross-sectional designs of the previously mentioned studies and the absence of strength measures in the current study limit our ability to tease out whether or not the shorter COP-eCOM differences are *primary* deviations related to a lack of neuromuscular control or *secondary compensation* of the disease. Future studies that incorporate longitudinal designs and strength measures will ultimately be required to delineate these underlying factors.

A limitation of the study is that we only examined one walking speed, one turning direction, and one turning angle. Specifically, the participants were instructed to walk at their self-selected, comfortable pace and then turn to their dominant side at the stanchions and continue walking in the new direction. Although the instructions for participants to walk at their “self-selected, comfortable pace” were instituted in order to assess participants during their most frequently utilized walking speeds, these instructions are likely to have increased the variability of walking speed across subjects. It is not clear, however, if increased walking-speed variability would also increase the variability of our primary outcome variable, COP-eCOM, because participants may select a safe turning strategy that preserves COP-eCOM distance, independent of walking speed. Moreover, individuals will often have to modify their movement speed (either slowing or speeding-up) during ADLs in response to external/environmental conditions (e.g., weather, traffic lights, ground/floor frictional characteristics, obstacles, etc.). Thus, future studies should examine the effects of *speed* on postural control during turning in persons with PD, and include trials “as fast as possible”, “as safe as possible”, and at other predetermined speeds. Additionally, in order to navigate successfully, people must turn both right and left and negotiate a variety of turning angles (although the majority of turns experienced during ADLs are between 76° and 120°) [21]. These additional directions and turning angles should also be examined in future study designs.

This study also did not examine the influence of medication on turning behavior and COP-eCOM. Participants that had pharmacological treatment were stable with no fluctuations of PD symptoms and tested while they were on their routine therapy. At the time of testing, none of the participants exhibited dyskinesia, dystonia, or other signs of involuntary movement. Thus, whether or not COP-eCOM distances would have been different had the participants not been on their routine therapy cannot be inferred from the current investigation. In a recent report, Hong and Earhart reported that although medication significantly improved UPDRS scores and walking velocity, it did not statistically significantly alter turning performance [30]. The authors went on, however, to report that “there was evidence for [turning] improvements particularly with respect to the amplitudes of relative rotation between segment rotations with effect sizes ranging from 0.42 to 0.70.” They noted that their “…results suggest that only certain features of impaired turning may be responsive to anti-Parkinson's medication.” The participants in the Hong and Earhart study were older and had more advanced PD than participants in the current study—making extrapolation of their findings to the current investigation difficult. We hypothesize, however, that medication effects on turning in persons with *early* PD will be less evident. Additional studies investigating the influence of medication on COP-eCOM during turning will be needed in persons with EPD to test this hypothesis.

Despite these limitations, the results of the current study provide important additional evidence that functional impairments can be detected even in the early stages of the disease, when clinical signs of gait disturbance are often absent [5, 31]. Taken together, these reports suggest that identifying the movement limitations associated with EPD requires examination of more complex tasks that increase the challenge to the neuromuscular system, such as turning and gait initiation. The findings also suggest that the peak COP-eCOM distance generated during turning activities may be a useful index for quantifying disease severity and intervention effectiveness. In order to determine whether the postural control strategies during step turning are sensitive to disease severity, additional studies that examine individuals across a broader range of disease severity will be necessary. Additionally, studies will be needed to delineate the influence of rehabilitation interventions on postural control during turning in persons with EPD.

## 5. Conclusion

Compared to HC participants, persons with EPD altered their postural control strategies (shorter distance between the COP and the eCOM) during the step turn. Persons with EPD appear to decrease their overall movement amplitude (i.e., COM displacement, velocity) suggesting that dynamic postural control during turning is altered even in the early stages of PD.

## Figures and Tables

**Table 1:** Mean and standard deviation of participant's characteristics.

	Group		
	EPD (*n* = 15)	HC (*n* = 10)	Differences (95% CI)
Age (yr)	62 (9.1)	60 (8.5)	2 (−5.49; 9.49)
Height (m)	1.68 (0.07)	1.72 (0.09)	−0.04 (−0.11; 0.03)
Weight (kg)	68.9 (12.1)	74.8 (17.2)	−5.9 (−17.99; 6.19)
Approach gait velocity (m/s)	1.35 (0.14)	1.46 (0.14)	−0.11 (−0.23; 0.01)

Mean (Standard Deviation).

EPD: persons with early-stage Parkinson's disease.

HC: healthy control participants.

**Table 2:** Dosage of Parkinson's medications.

Participant number	Medication	Dosage	Frequency
P01	Levodopa/carbidopa	25–100 mg	3x/day
P02	De Novo		
P03	Pramipexole	1 mg	3x/day
	Rasagiline	1 mg	1x/day
	Levodopa/carbidopa	50–200 mg	3x/day
P04	De Novo		
P05	Pramipexole	1.5 mg	3x/day
	Selegiline	5 mg	2x/day
P06	Rasagiline	1 mg	1x/day
	Amantadine	100 mg	2x/day
P07	De Novo		
P08	Rasagiline	1 mg	1x/day
	Trihexyphenidyl	4 to 6 mg	1x/day
P09	Levodopa/carbidopa	150 mg	3x/day
P10	Pramipexole	0.75 mg	3x/day
	Rasagiline	1 mg	1x/day
P11	Pramipexole	1.5 mg	3x/day
	Rasagiline	1 mg	1x/day
P12	Levodopa/carbidopa	25–100 mg	4x/day
P13	Levodopa/carbidopa	25–100 mg	3x/day
	Rasagiline	0.5 mg	1x/day
P14	Pramipexole	0.5 mg	3x/day
P15	Pramipexole	1.5 mg	3x/day
	Selegiline	5 mg	2x/day
	Trihexyphenidyl	2 mg	3x/day
